# Do doctors practice what they preach? The wellbeing and lifestyle habits of primary health care physicians in Bahrain

**DOI:** 10.1186/1753-6561-9-S1-A3

**Published:** 2015-01-14

**Authors:** SM Borgan, ZA Marhoon, MA Ebrahim, MA Almuqamam

**Affiliations:** 1 Royal College of Surgeons in Bahrain, P.O. Box 15503, Adilya, Bahrain

## Background

Lifestyle habits of physicians are of paramount importance for two reasons. Firstly, they influence and direct the physician's own health. Secondly, it has been shown that these habits have implications on patients' care. There is limited information on the lifestyle habits and wellbeing of physicians in Bahrain. Therefore, we set out lifestyle habits and the general wellbeing of practicing primary health care physicians in Bahrain.

## Methods

A cross sectional study. An anonymous self-administered questionnaire assessing wellbeing and lifestyle habits was distributed to a random sample of 175 out of 320 primary health care physicians who practice in all 27 health centres around Bahrain. We performed descriptive analysis for all variables. Parametric test (t-test and ANOVA) and Pearson two-tailed test were used to test the association between variables were appropriate.

## Results

One hundred fifty-two physicians agreed to participate in the study. Female physicians made up two thirds of the sample. The majority are of Bahraini nationality with a mean age of 45 (SD=10). The most prevalent known health conditions are hyperlipidaemia (25%), hypertension (20%) and diabetes (11%). Only 30% of the physicians report a 30 minute exercise in a usual week; of those, 13% exercise for 5 days or more. The majority of physicians report walking as their main exercise form. Concerning nutrition, 41% have three main meals every day. Forty seven percent of physicians consume fast-food meals during the week while a similar percentage drinks at least one carbonated beverage each day. Regarding smoking and alcohol consumption, 98% report never drinking alcohol ever, while tobacco smoking is used by 10%, with 6% of the sample smoking waterpipe. The mean sleeping time is 6 hours a day (SD=1). The average body-mass-index is 28 (SD=5) with 39% being overweight and 33% in the obese range. Body-mass-index is related to 6 variables: Older age, Male gender, less sleep time and being diagnosed with diabetes, hypertension and hyperlipidaemia.

## Conclusions

There is a clear pattern of unfavourable lifestyle habits as well as a high prevalence of hyperlipidaemia, hypertension and obesity among primary health care physicians in Bahrain. Institutions are encouraged to further enlighten physicians on the importance of living healthy lifestyles.

